# Factors associated with initiation of antiretroviral therapy in the advanced stages of HIV infection in six Ethiopian HIV clinics, 2012 to 2013

**DOI:** 10.7448/IAS.19.1.20637

**Published:** 2016-04-22

**Authors:** Denis Nash, Olga Tymejczyk, Tsigereda Gadisa, Sarah Gorrell Kulkarni, Susie Hoffman, Muluneh Yigzaw, Batya Elul, Robert H Remien, Maria Lahuerta, Shalo Daba, Wafaa El Sadr, Zenebe Melaku

**Affiliations:** 1 Department of Epidemiology, Mailman School of Public Health, Columbia University, New York, NY, USA; 2 Department of Epidemiology, City University of New York, School of Public Health, New York, NY, USA; 3 HIV Center for Clinical and Behavioral Studies, New York, NY, USA; 4 ICAP, Mailman School of Public Health, Columbia University, New York, NY, USA; 5 Oromia State Regional Bureau of Health, Addis Ababa, Ethiopia

**Keywords:** HIV-positive adults, antiretroviral therapy initiation, tuberculosis treatment, Ethiopia, antiretroviral therapy guidelines, implementation science

## Abstract

**Introduction:**

Most HIV-positive persons in sub-Saharan Africa initiate antiretroviral therapy (ART) with advanced infection (late ART initiation). Intervening on the drivers of late ART initiation is a critical step towards achieving the full potential of HIV treatment scale-up. This study aimed to identify modifiable factors associated with late ART initiation in Ethiopia.

**Methods:**

From 2012 to 2013, Ethiopian adults (*n*=1180) were interviewed within two weeks of ART initiation. Interview data were merged with HIV care histories to assess correlates of late ART initiation (CD4+ count <150 cells/µL or World Health Organization Stage IV).

**Results:**

The median CD4 count at enrolment in HIV care was 263 cells/µL (interquartile range (IQR): 140 to 390) and 212 cells/µL (IQR: 119 to 288) at ART initiation. Overall, 31.2% of participants initiated ART late, of whom 85.1% already had advanced HIV disease at enrolment. Factors associated with higher odds of late ART initiation included male sex (vs. non-pregnant females; adjusted odds ratio (aOR): 2.02; 95% CI: 1.50 to 2.73), high levels of psychological distress (vs. low/none, aOR: 1.96; 95% CI: 1.34 to 2.87), perceived communication barriers with providers (aOR: 2.42, 95% CI: 1.24 to 4.75), diagnosis via provider initiated testing (vs. voluntary counselling and testing, aOR: 1.47, 95% CI: 1.07 to 2.04), tuberculosis (TB) treatment prior to ART initiation (aOR: 2.16, 95% CI: 1.43 to 3.25) and a gap in care of six months or more prior to ART initiation (aOR: 2.02, 95% CI: 1.10 to 3.72). Testing because of partner illness/death (aOR: 0.64, 95% CI: 0.42 to 0.95) was associated with lower odds of late ART initiation.

**Conclusions:**

Programmatic initiatives promoting earlier diagnosis, engagement in pre-ART care, and integration of TB and HIV treatments may facilitate earlier ART initiation. Men and those experiencing psychological distress may also benefit from targeted support prior to ART initiation.

## Introduction

Although HIV care services have been increasingly scaled up [1], most HIV-positive persons in sub-Saharan Africa start treatment only after developing advanced infection, which leads to high early mortality [2], complex and expensive clinical management [3], blunted immune response [4] and missed opportunities to prevent HIV transmission [5]. The problem of late antiretroviral therapy (ART) initiation in sub-Saharan Africa has improved only slightly since the start of HIV scale-up in the region [6,7], making it important to identify its determinants, as well as necessary programmatic adjustments and policy changes.

Studies of factors associated with advanced HIV infection prior to ART initiation (i.e. *at diagnosis and/or presentation to care*) in sub-Saharan Africa have found that sex, pregnancy, age, family composition, living arrangements, education level, employment status, competing priorities, disclosure status, emotional health and alcohol use were important correlates [8–13]. Patients’ perceptions of stigma, medication side effects and healthcare access barriers may also contribute [8,10]. Demographic and clinical factors associated with a higher likelihood of advanced HIV disease *at ART initiation* have included male sex, being already beyond the point of ART eligibility at the time of enrolment into HIV care, TB treatment at ART initiation and a ≥12 month gap in care between enrolment and ART initiation [7,14]. A qualitative study from Uganda additionally reported patient concerns about stigma, lack of confidentiality, low social support and misconceptions about ART as barriers to ART initiation [15]. However, most studies have not examined factors beyond those routinely collected in HIV clinical records or the role of the pre-ART phase of care, limiting the ability to inform the development of interventions.

Ethiopia, with an estimated HIV prevalence among adults of 1.3% (590,000 persons) and 4.2% in urban areas [1,16], began scaling up comprehensive HIV services (including ART) in 2005. In 2012, however, only 68% of adult Ethiopians eligible for ART were receiving it [1]. In order to help maximize the individual and public health benefit from the treatment eligibility guideline expansion [17], the present study was designed to identify the major drivers of late ART initiation, particularly those that are amenable to intervention.

## Methods

### Study setting

We enrolled 1180 patients initiating ART at six HIV clinical care sites in the Oromia region of Ethiopia, and part of the Ethiopian National ART Program, under the jurisdiction of the Oromia Regional Health Bureau. The combined catchment areas of these clinics cover 9.3 million people, with background HIV prevalence estimates ranging from 3.2 to 11.5%, according to antenatal clinic (ANC) data at the sites. The clinics have been supported by the Ethiopian Ministry of Health with technical assistance from ICAP at Columbia University since 2005, via funding from the President's Emergency Plan for AIDS Relief. All six sites are secondary health facilities in urban areas, with on-site CD4+ testing and ART pharmacies. All offer voluntary counselling and testing (VCT), conduct provider initiated counselling and testing (PITC) and have ANC and labour and delivery wards. In January 2013, during the course of this study, the national guidelines for ART initiation were expanded from World Health Organization (WHO) Stage IV or CD4+ count <200 cells/µL or WHO Stage III with CD4+ <350 cells/µL to include all patients with CD4+ <350 cells/µL.

### Participant recruitment and data collection

#### Interviews

All non-institutionalized patients aged 18 years and older initiating ART between June 2012 and April 2013 were eligible for inclusion in the study. Such persons were referred by providers to the study staff on the day of ART initiation. Study staff administered the structured questionnaire (lasting 45 to 60 minutes) with questions related to psychosocial factors and HIV care. The interviews were conducted within two weeks of the day of ART initiation, and participants were given a snack and money for transportation home from the clinic (20 Ethiopian birr, or approximately 1 USD). Among the eligible patients referred to the study, 95% consented and completed the interview.

#### Routine clinical information

Data obtained from patients at the time of HIV care enrolment and each subsequent clinic visit were recorded on national forms. In accordance with routine clinic procedures, clerks entered medical chart data into electronic databases. Assessments of data entry completeness and accuracy were done regularly. Interview data were linked with clinic medical records for the period between HIV care enrolment and ART initiation and then de-identified for analysis.

### Measures

#### Outcome

Advanced HIV infection at ART initiation was defined as having a CD4+ count <150 cells/µL or WHO Stage IV. CD4+ count or WHO stage at ART initiation included those measurements taken three months before or one month after ART initiation. In instances where CD4+ count or WHO stage were missing in the above window, we used the highest stage preceding ART initiation and any prior CD4+ count <150 cells/µL.

#### Psychosocial variables

Questionnaire design and variable selection were guided by the framework of health service use developed by Aday and Andersen [18]. Predisposing factors (i.e. pre-existing propensity to access care) examined included sex, age, education, relationship status, having ever had children, alcohol use, psychological distress, enacted and internalized stigma, and history of holy water use for HIV. Alcohol use frequency was divided into high (at least twice a week), moderate (at least monthly) and low/none (never or not in the last three months). Psychological distress was assessed through the Kessler-10 scale, with three categories of distress: high (score of 30 to 50), mild/moderate (20 to 29) and low/none (10 to 19) [19]. Internalized and enacted stigma measures (alpha=0.99 with statements such as “you felt completely worthless,” and alpha=0.94 with statements such as “someone scolded you”) had answer options ranging from “most of the time” to “never” and were drawn from the instrument developed by Holzemer *et al*. [20]. For analysis, average scores on internalized stigma were grouped into tertiles and those on enacted stigma into “any stigma” or “none,” based on the respective score distributions. We also examined beliefs related to ART through a set of 10 items (alpha=0.74), for example, “AIDS no longer kills everyone because of the ARV medicines,” with responses on a 4-point Likert scale (ranging from 1, “strongly disagree,” to 4, “strongly agree”). Mean scores were categorized into high accuracy (if the score corresponded to “agree” or “strongly agree”) and low accuracy [21].

Enabling factors (i.e. means available to access care) examined included urban/rural residence; food insecurity; knowing someone on ART; HIV status disclosure; social support (9 questions; Cronbach's alpha=0.92, adapted from previous work by Wortman [22] with questions such as “Would someone be available to talk to you if you were upset, nervous or depressed?”); and communication barriers with providers (difficulties understanding information about HIV care or ART side effects).

Having been tested for HIV because of partner's sickness, death or HIV diagnosis was examined within the “need” component of the framework.

#### Correlates from routine clinical information

CD4+ count at enrolment in HIV care was defined as the earliest recorded CD4+ measurement in the first six months in care. A gap in care was defined as not having had a clinic visit for at least six months prior to ART initiation. Enrolment point of entry captured the source of the patient's referral to HIV care. Receipt of TB treatment at any point prior to ART initiation was also recorded.

### Statistical methods

We compared the distribution of key sample characteristics between male and female participants using chi-square and Mann-Whitney tests of statistical significance. Potential correlates of late ART initiation were examined through bivariate and multivariable logistic regression. To assess added information on correlates of late ART initiation that may be gained from psychosocial data, two separate regression models were constructed using only psychosocial data and then combining them with routine clinical variables. Multivariable regression models initially included all variables that had *p*-values <0.20 in bivariate analyses. Covariates with the highest *p*-values were eliminated through backward stepwise regression until all remaining variables had *p*-values <0.05. Eliminated variables were then added individually back to this model in case of negative confounding (i.e. associations observed only in the presence of other variables). Sex and age group were retained in multivariable models in order to produce sex and age-adjusted odds ratios. Sex-specific models were also constructed to elucidate possible differences in correlates by gender. Median CD4+ counts at ART initiation were plotted by month and sex to assess the trend over time and possible impact of expanded guidelines on ART uptake. Analyses were conducted in SAS 9.3 (SAS Institute, Cary, NC, USA) using conditional logistic models with clinical sites as strata.

### Ethical considerations

The study was approved by the Institutional Review Boards of the Oromia Regional Health Bureau, Columbia University Medical Center and the City University of New York. Written, informed consent for the interview and extraction of medical record data was obtained from each patient prior to study enrolment.

## Results

### Characteristics of persons initiating ART

The majority of participants were women (61.2%) and the median age was 34 (interquartile range (IQR): 28 to 40). Most patients were Ethiopian Orthodox (69.9%), urban residents (78.0%) and had no more than primary school education (71.8%). Women were more likely than men to have received no education (39.1% vs. 20.1%, *p*<0.001). More than half of the participants were in a relationship at the time of ART initiation and a quarter had been widowed. The largest proportion (55.7%) was first diagnosed with HIV in a PITC setting (Table 1).

**Table 1:** Characteristics of patients initiating ART, by sex, at six Ethiopian clinics (June 2012 to April 2013)

	Total						
						
	Sample		Men		Women		
				
	*n*	%	*n*	%	*n*	%	*p*^a^
Total	1180	100.0	458	38.8	722	61.2	–
Sex							
Male	458	38.8	–	–	–	–	–
Female, not pregnant	669	56.7	–	–	–	–	
Female, pregnant	53	4.5	–	–	–	–	
Age							
Median (IQR)	34 (28 to 40)		37 (32 to 43)		30 (26 to 38)		<0.001
18 to 29	374	31.7	78	17.0	296	41.0	<0.001
30 to 39	482	40.8	205	44.8	277	38.4	
40 to 49	238	20.2	131	28.6	107	14.8	
50 +	86	7.3	44	9.6	42	5.8	
Religion							
Ethiopian Orthodox	825	69.9	323	70.5	502	69.5	0.549
Protestant	239	20.3	85	18.6	154	21.3	
Muslim	108	9.2	47	10.3	61	8.4	
Other Christian	8	0.7	3	0.7	5	0.7	
Highest education level							
No school	374	31.7	92	20.1	282	39.1	<0.001
Primary school or vocational/other	484	41.0	206	45.0	278	38.5	
Secondary school	239	20.3	111	24.2	128	17.7	
University	83	7.0	49	10.7	34	4.7	
Area of residence							
Urban area	920	78.0	335	73.1	585	81.0	0.002
Rural area	259	21.9	122	26.6	137	19.0	
Unknown	1	0.1	1	0.2	0	0.0	
Relationship status							
In a relationship	673	57.0	309	67.5	364	50.4	<0.001
Not in a relationship	507	43.0	149	32.5	358	49.6	
Ever widowed							
Yes	293	24.8	75	16.4	218	30.2	<0.001
No	874	74.1	377	82.3	497	68.8	
Unknown	13	1.1	6	1.3	7	1.0	
Original diagnosis unit							
PITC	647	54.8	245	53.5	402	55.7	0.763
VCT	513	43.5	205	44.8	308	42.6	
Unknown	20	1.7	8	1.7	12	1.7	
Site							
Ambo Hospital	241	20.4	109	23.8	132	18.3	0.027
Bishoftu Hospital	309	26.2	133	29.0	176	24.4	
Fitche Hospital	166	14.1	53	11.6	113	15.7	
Goba Hospital	129	10.9	45	9.8	84	11.6	
Nekemte Hospital	194	16.4	70	15.3	124	17.2	
Shashemene Hospital	141	11.9	48	10.5	93	12.9	

ART, antiretroviral therapy; IQR, interquartile range; PITC, provider-initiated testing and counselling; VCT, voluntary counselling and testing

a chi-squared test for categorical variables, Mann-Whitney *U* test for continuous variables.

### Clinical and immunological characteristics at enrolment and ART initiation

Of the 97.4% of participants with available CD4+ counts and/or WHO stage at enrolment in HIV care, 27.3% had enrolled in HIV care with advanced HIV infection (CD4 <150 cells/µL or WHO Stage IV). The median enrolment CD4+ count among the 94.0% of participants with available data was 263 cells/µL (IQR: 140 to 390), with significantly higher values among women (median=296; IQR: 172 to 423) than men (median=206; IQR: 108 to 344, *p*<0.001). Overall, 47.8% of participants were already beyond the point of ART eligibility at enrolment in HIV care, including 57.9% of men and 41.4% of women (*p*<0.001). Median time between enrolment in care and ART initiation was 2.9 months (IQR: 15 days to 2.6 years). This period was significantly shorter among men than women (median 1.1 months vs. 5.3 months, respectively; *p*<0.001). At ART initiation, 31.2% of participants were classified as having late-stage HIV infection, most of whom (85.1%) already had advanced HIV disease at enrolment in care. Among the 91.2% of participants with available data, the median CD4+ count at treatment initiation was 212 cells/µL (IQR: 119 to 288) and was significantly higher among women than men (median=232; IQR: 152 to 302; vs. median=178; IQR: 98 to 251, respectively; *p<*0.001). After HIV care enrolment and prior to ART initiation, 12.7% of patients underwent TB treatment (Table 2). Among these patients, the median delay between initiation of TB treatment and ART was 27 days (IQR: 14 to 97 days), with 65% starting ART within eight weeks.

**Table 2:** Clinical and immunological characteristics at enrolment in care and ART initiation at six Ethiopian clinics (June 2012 to April 2013)

	Enrolment in care			ART initiation		
		
	*n* (%)	% among men	% among women	*n* (%)	% among men	% among women
Total	1180 (100)			1180 (100)		
Advanced HIV disease^a^						
Data available	1149 (97.4)	98.0	96.8	1180 (100)	100	100
Yes	313 (27.3)	38.3	20.1	368 (31.2)	43.2	23.6
No	835 (72.7)	61.7	79.9	812 (68.8)	56.7	76.4
CD4+ count, cells/µL						
Data available	1109 (94.0)	6.1	6.0	1076 (91.2)	93.9	89.5
Median (IQR)	263 (140 to 390)	206 (108 to 344)	296 (172 to 423)	212 (119 to 288)	178 (98 to 251)	232 (152 to 302)
Clinical WHO disease stage						
Data available	1136 (96.3)	96.9	95.7	1177 (99.7)	99.8	99.6
Stage I	381 (33.6)	22.5	40.6	257 (21.8)	14.9	26.2
Stage II	307 (27.0)	30.2	25.0	335 (28.5)	28.5	28.5
Stage III	418 (36.8)	43.9	32.2	540 (45.9)	52.7	41.6
Stage IV	30 (2.6)	3.4	2.2	45 (3.8)	3.9	3.8
ART eligibility at enrolment^b^						
Not eligible	585 (49.6)	40.1	55.5	–	–	
Eligible	564 (47.8)	57.9	41.4	–	–	
Unknown	31 (2.6)	2.0	3.1	–	–	
History of TB treatment in HIV care						
Yes	–	–		150 (12.7)	15.9	10.7
No	–	–		1030 (87.3)	84.1	89.3
Time between diagnosis and ART initiation						
Median (IQR)	8.3 months (1.1 months to 3.4 years)					
Men	3.6 months (27 days to 2.4 years)					
Women	1.1 years (1.2 months to 4 years)					
Time between enrolment in care and ART initiation						
Median (IQR)	2.9 months (15 days to 2.6 years)					
Men	1.1 months (14 days to 1.4 months)					
Women	5.3 months (18 days to 3.1 years)					

ART, antiretroviral therapy; IQR, interquartile range; TB, tuberculosis; WHO, World Health Organization

a advanced HIV disease at enrolment or ART initiation is defined as having a CD4+ cell count <150 cells/µL or WHO Stage IV

b eligibility based on national Ethiopian HIV guidelines.

**Figure 1:**
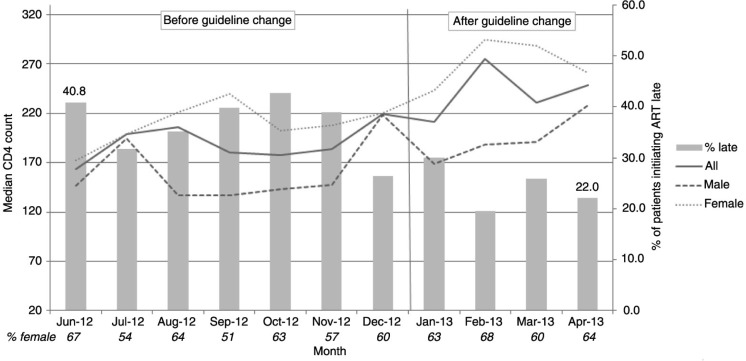
Median CD4+ count at antiretroviral therapy (ART) initiation and proportion of patients initiating ART late, by month and sex.

During the study period, there was an appreciable increase in the median CD4+ count at ART initiation. An increase in CD4+ counts was observed among both men (average of 30 cells) and women (average of 78 cells) after the January 2013 guideline expansion (*p*=0.003 and *p*<0.001, respectively). The proportion of patients starting treatment with advanced HIV infection decreased significantly, from 40.8% in June 2012 to 22.0% in April 2013 (*p*_trend_<0.001; Figure 1).

### Factors associated with late ART initiation

Table 3 presents bivariate associations, a multivariable model including only the psychosocial variables and a final multivariable model incorporating the clinical variables.

**Table 3:** Bivariate and multivariable associations between participants’ characteristics and late ART initiation^a^

		Bivariate	Multivariable Model 1 (*N*=1171)^b^	Multivariable Model 2 (*N*=1174)^c^
				
	*n*	OR (95% CI)	aOR (95% CI)	aOR (95% CI)
Predisposing factors				
Sex (ref: non-pregnant women, *n*=669)				
Men	458	2.34 (1.81 to 3.03)	2.29 (1.73 to 3.02)	2.02 (1.50 to 2.73)
Pregnant women	53	0.44 (0.19 to 1.00)	0.41 (0.18 to 0.97)	0.53 (0.21 to 1.33)
Age at ART initiation (ref: 18 to 29, *n*=374)				
30 to 39	482	1.63 (1.21 to 2.19)	1.27 (0.92 to 1.75)	1.25 (0.88 to 1.77)
40 to 49	238	1.29 (0.90 to 1.85)	0.91 (0.61 to 1.35)	0.92 (0.60 to 1.40)
50 +	86	1.14 (0.67 to 1.93)	0.72 (0.41 to 1.26)	0.60 (0.33 to 1.10)
Education level (ref: primary school or vocational/other, *n*=484)				
No school	374	0.78 (0.57 to 1.05)		
Secondary school	239	1.09 (0.78 to 1.51)		
University	83	1.16 (0.71 to 1.91)		
Relationship status (ref: in a relationship, *n*=673)				
Not in a relationship	507	1.15 (0.90 to 1.48)		
Has ever had children (ref: no, *n*=253)				
Yes	926	0.78 (0.58 to 1.05)		
Alcohol use (ref: low or none, *n*=916)				
High	162	1.59 (1.12 to 2.26)		
Moderate	102	1.06 (0.68 to 1.67)		
Psychological distress (ref: low or none, *n*=480)				
Medium	348	1.93 (1.41 to 2.66)	1.76 (1.27 to 2.46)	1.61 (1.12 to 2.31)
High	347	2.91 (2.08 to 4.07)	2.70 (1.90 to 3.85)	1.96 (1.34 to 2.87)
Enacted stigma (ref: none, *n*=983)				
Any	192	1.05 (0.74 to 1.48)		
Internalized stigma (ref: top tercile/least stigma, *n*=469)				
Bottom tercile (=most stigma)	395	1.44 (1.06 to 1.94)		
Middle tercile	310	1.24 (0.90 to 1.70)		
History of holy water use for HIV (ref: no, *n*=840)				
Yes	189	0.69 (0.48 to 0.99)		
Treatment beliefs (ref: low accuracy, *n*=118)				
High accuracy	1061	0.86 (0.56 to 1.32)		
Enabling factors				
Area of residence (ref: rural, *n*=259)				
Urban	920	0.79 (0.59 to 1.07)		
Trouble satisfying food needs in last year (ref: never, *n*=422)				
Sometimes	502	0.91 (0.68 to 1.22)		
Often	252	0.84 (0.59 to 1.19)		
Knows someone on ART (ref: no, *n*=368)				
Yes	809	0.73 (0.56 to 0.95)	0.77 (0.57 to 1.04)	
Number of people disclosed to (ref: 0, *n*=167)				
1	422	0.79 (0.55 to 1.15)	1.00 (0.67 to 1.49)	
≥ 2	591	0.57 (0.40 to 0.82)	0.69 (0.46 to 1.02)	
Social support (ref: bottom quarter/least support; *n*=298)				
Second quarter	305	0.91 (0.64 to 1.28)		
Third quarter	292	0.84 (0.59 to 1.19)		
Top quarter (= most support)	285	0.90 (0.62 to 1.30)		
Communication barriers with providers (ref: no, *n*=1127)				
Yes	53	2.27 (1.30 to 3.97)	2.43 (1.32 to 4.48)	2.42 (1.24 to 4.75)
Perceived need				
Tested for HIV because partner sick/dead/HIV+ (ref: no, *n*=930)				
Yes	250	0.38 (0.27 to 0.55)	0.47 (0.32 to 0.69)	0.64 (0.42 to 0.95)
Clinical characteristics				
Enrolment point of entry (ref: VCT, *n*=493)				
PMTCT	45	0.31 (0.11 to 0.88)	–	0.56 (0.18 to 1.72)
PITC	491	1.80 (1.36 to 2.39)	–	1.47 (1.07 to 2.04)
Other, including TB clinic	32	2.15 (1.03 to 4.49)	–	1.85 (0.73 to 4.71)
Unknown	119	2.22 (1.45 to 3.42)	–	1.70 (1.04 to 2.77)
History of TB treatment in HIV care (ref: no, *n*=1030)				
Yes	150	2.93 (2.07 to 4.16)	–	2.16 (1.43 to 3.25)
Gap in care of six months or more prior to ART (ref: no, *n*=260)				
Yes	260	2.41 (1.35 to 4.33)	–	2.02 (1.10 to 3.72)
Initiated ART during first six months in care	659	11.64 (7.04 to 19.25)	–	8.98 (5.34 to 15.08)

aOR, adjusted odds ratio; ART, antiretroviral therapy; OR, odds ratio; VCT, voluntary counselling and testing; PMTCT, prevention of mother-to-child transmission; PITC, provider-initiated testing and counselling; TB, tuberculosis

a late ART initiation is defined as having a CD4+ cell count <150 cells/µL or WHO Stage IV. Analyses account for site-level clustering. Statistically significant associations (*p*<0.05) in bold

b multivariable Model 1 includes only psychosocial factors

c multivariable Model 2 is final, combining psychosocial and clinical factors.

#### Psychosocial variables

In the final multivariable model, men had twice the odds of late ART initiation as non-pregnant women (aOR: 2.02; 95% CI: 1.50 to 2.73). Psychological distress (high vs. low/none, aOR: 1.96; 95% CI: 1.34 to 2.87) and perceived communication barriers with providers (vs. no barriers, aOR: 2.42; 95% CI: 1.24 to 4.75) were associated with increased odds of late ART initiation, whereas testing for HIV because of a partner's death or illness (vs. not, aOR: 0.64; 95% CI: 0.42 to 0.95) was associated with lower odds. In the multivariable model containing only psychosocial variables, patients who knew someone on ART (vs. not, aOR: 0.77; 95% CI: 0.57 to 1.04) and those who had disclosed their status to two or more people (vs. 0, aOR: 0.69; 95% CI: 0.46 to 1.02) were also marginally less likely to initiate treatment with advanced HIV disease, but these associations were no longer significant after adjustment for clinical factors.

#### Clinical variables

Participants referred to HIV care from PITC (vs. VCT, aOR: 1.47; 95% CI: 1.07 to 2.04) and those with history of TB treatment while in HIV care (vs. no treatment, aOR: 2.16; 95% CI: 1.43 to 3.25) had higher odds of initiating treatment with advanced HIV infection. Having a gap in pre-ART care of six months or more was also associated with late ART initiation (aOR: 2.02; 95% CI: 1.10 to 3.72), as was initiation in the first six months after enrolment in care (aOR: 8.98; 95% CI: 5.34 to 15.08). Inclusion of the gap in care variable eliminated the disclosure variable from the final model (Table 3).

#### Sex-specific models

When psychosocial and clinical data were analyzed separately for men and women, sex differences included marginally lower odds of late ART initiation by men if they had disclosed their HIV status to at least two people (vs. none, aOR: 0.61; 95% CI: 0.36 to 1.05) and higher odds of late ART initiation if referred to HIV care from PITC settings (vs. VCT, aOR: 1.96; 95% CI: 1.21 to 3.18). In contrast, women had higher odds of late treatment initiation if they reported experiencing communication barriers with providers (vs. none, aOR: 2.51; 95% CI: 1.10 to 5.75) (models not shown).

## Discussion

In our study, conducted with a large sample of patients initiating ART in a mature national HIV programme in East Africa, 31.2% of patients started ART with advanced HIV infection (CD4+ <150 cells/µL or WHO Stage IV), of whom 85.1% already had advanced HIV infection at the time of enrolment in care. This finding underscores the need to promote and expand testing coverage in the community to provide opportunities for earlier diagnosis with timely linkage to care, particularly for men. Additionally, we identified a number of factors that could represent potentially important targets for interventions aimed at reducing the persistently high rates of late ART initiation.

In multivariable analysis, consistent with prior research [7,14], male sex was a strong correlate of late ART initiation, with men having twice the odds of starting treatment with advanced HIV infection as non-pregnant women. This disparity is increasing over time [7] and has been at least partly attributed to women's increasing access to PMTCT services [23,24], as well as differences in health-seeking behaviour resulting in delayed diagnosis and care entry among men [25].

Patients with a gap in care of six months or greater prior to ART initiation had twice the odds of initiating treatment with advanced HIV infection. This finding is consistent with the results of a recent large-scale analysis of data from sub-Saharan Africa conducted by our team [7]. Others have noted that fewer than a third of patients not eligible for treatment at the time of enrolment in care are retained in pre-ART care in the region [26]. In one study conducted in Ethiopia, those with less advanced HIV infection were more likely to be lost to follow-up prior to ART initiation than those with advanced HIV infection [12]. Patients enrolling in care at the early stages of HIV infection may therefore be more likely to remain engaged in care where there are services that could be perceived as beneficial to them such as free co-trimoxazole [27] and time-saving clinic-level efficiencies, such as reliable point-of-care CD4+ testing [28].

Additional support may be beneficial for patients undergoing TB treatment in HIV care, who had twice the odds of late ART initiation as those who did not undergo TB treatment; an association previously also reported elsewhere in sub-Saharan Africa [7]. Although TB disease is more common among persons with advanced HIV-related immunosuppression, both the disease itself and TB therapy can each be markers of and risk factors for late ART initiation. TB disease can accelerate CD4+ decline [29] and uptake of treatment guidelines in clinical practice can take time [30]. Although WHO guidelines recommend that TB treatment be started first, with ART added as soon as possible during the first eight weeks of TB therapy [17,31], only 65% of TB patients in our study initiated ART within that period. Concerns about drug interactions, additional side effects, immune reconstitution inflammatory syndrome and high pill burden often cause ART initiation to be delayed until TB treatment has been completed, despite evidence that integration of TB and HIV treatment may successfully extend AIDS-free survival of severely immunocompromised patients [32].

Alongside clinical variables, several psychosocial factors were identified as significant correlates of late ART initiation. The marginal association between disclosure of HIV status to at least two people and lower odds of late ART initiation suggests that practical and emotional support potentially enabled by disclosure may facilitate engagement in care and timely treatment initiation. A similar observation was previously reported in another Ethiopian study, where non-disclosure of HIV status was associated with late presentation to HIV care [10]. Although facilitation of disclosure and testing of family members, mainly partners, is recognized by HIV care providers as a critical element of pre-ART care, disclosure appears to be discussed less frequently with patients after initial enrolment visits [33], perhaps because most patients have by then disclosed their status to at least one person. However, our findings suggest that it may be worthwhile for clinic staff to continue encouraging disclosure, especially among men, even after a patient has disclosed to one other person. That the disclosure variable was eliminated from our final multivariable model when the gap in care variable was introduced suggests that time since enrolment in care partially confounds the association between disclosure and late ART initiation [34], but also that poor engagement in care may be one of the ways that under-disclosure influences the risk of delayed treatment initiation.

Enacted and internalized stigma were not significantly associated with late ART initiation, consistent with a prior ecological study from eight sub-Saharan African countries [35], but in contrast to qualitative research from the region [15]. Psychological distress, however, previously linked with late HIV diagnosis [8], was strongly associated with late ART initiation in our analysis. Although advanced HIV disease may be a cause of psychological distress, rather than the reverse, this association suggests that it is important for providers to screen for signs of distress at enrolment in HIV care and subsequent clinic visits, with referral to appropriate/available resources such as peer support or mental health services, where available.

Problems understanding HIV care providers, reported by nearly 5% of participants, can constitute a barrier to timely treatment initiation, especially among women, for whom this variable remained highly significant in final sex-specific models. This finding underscores the need for individualized, stage-appropriate counselling [36], as well as frequent assessments of patient understanding/comprehension during clinic visits.

The impact of gaps in care on late ART initiation may have been lessened by the January 2013 expansion of Ethiopia's national ART guidelines to include all patients with CD4+ counts ≤350 [17]. We observed a substantial decrease in the proportion of patients initiating treatment late and an increase in median CD4+ counts at initiation among both men and women during the study period (June 2012 to April 2013). This finding suggests that the expansion of ART initiation guidelines enabled the clinics to put more patients who were already diagnosed and engaged in pre-ART care on treatment earlier, before their health deteriorated further.

Major strengths of the study include the combination of longitudinal clinical data, including pre-ART care data, dating back to participants’ enrolment in care with self-reported information from interviews, as well as a high degree of data completeness. Our study also has some limitations. The clinics included in the study may not be representative of all settings in which Ethiopian patients receive HIV care and treatment, tempering our ability to further generalize our findings. A large proportion of persons initiating treatment with late-stage HIV already had advanced infection at enrolment in HIV care, and likely often at diagnosis, making it challenging to tease apart the correlates of late ART initiation from factors related to late enrolment or diagnosis. Additionally, our study did not include those patients who died prior to ART initiation, who may have had very advanced HIV disease. Finally, the cross-sectional nature of the interview relative to outcome measurement makes it difficult to sort out temporality of some exposures measured in the interviews in relation to the outcome.

## Conclusions

A substantial number of patients who initiated ART in our study already had advanced HIV infection at the time of enrolment into care, pointing to the need to expand testing coverage with timely linkage to care. Although the proportion of patients initiating ART late decreased over time in our study, many patients still started treatment with low CD4 counts (Figure 1). Patient sub-groups (such as men, those who have not disclosed their HIV status, those experiencing distress and those reporting communication difficulties with providers) may be at increased risk of late ART initiation. Additionally, elements of clinical histories (referral from PITC settings, gaps in pre-ART care, TB treatment) can be used by providers to identify patients who may be in need of targeted support in preparation for retention and timely ART initiation. Clinic-based programmatic initiatives promoting patient-centred, stage-appropriate counselling, engagement in pre-ART care and smoother integration of TB and HIV treatments may also facilitate more timely ART initiation.
